# Whole and Part Adaptive Fusion Graph Convolutional Networks for Skeleton-Based Action Recognition

**DOI:** 10.3390/s20247149

**Published:** 2020-12-13

**Authors:** Qi Zuo, Lian Zou, Cien Fan, Dongqian Li, Hao Jiang, Yifeng Liu

**Affiliations:** 1 School of Electronic Information, Wuhan University, Wuhan 430072, China; 2015301220073@whu.edu.cn (Q.Z.); fce@whu.edu.cn (C.F.); 2019202120070@whu.edu.cn (D.L.); jh@whu.edu.cn (H.J.); 2 National Engineering Laboratory for Risk Perception and Prevention (NEL-RPP), Beijing 100041, China; liuyifeng3@cetc.com.cn

**Keywords:** whole and part adaptive fusion, graph convolutional network, skeleton-based human action recognition

## Abstract

Spatiotemporal graph convolution has made significant progress in skeleton-based action recognition in recent years. Most of the existing graph convolution methods take all the joints of the human skeleton as the overall modeling graph, ignoring the differences in the movement patterns of various parts of the human, and cannot well connect the relationship between the different parts of the human skeleton. To capture the unique features of different parts of human skeleton data and the correlation of different parts, we propose two new graph convolution methods: the whole graph convolution network (WGCN) and the part graph convolution network (PGCN). WGCN learns the whole scale skeleton spatiotemporal features according to the movement patterns and physical structure of the human skeleton. PGCN divides the human skeleton graph into several subgraphs to learn the part scale spatiotemporal features. Moreover, we propose an adaptive fusion module that combines the two features for multiple complementary adaptive fusion to obtain more effective skeleton features. By coupling these proposals, we build a whole and part adaptive fusion graph convolution neural network (WPGCN) that outperforms previous state-of-the-art methods on three large-scale datasets: NTU RGB+D 60, NTU RGB+D 120, and Kinetics Skeleton 400.

## 1. Introduction

As one of the most significant parts of computer vision, human action recognition has had a wide range of applications in recent years, such as human-machine interaction, intelligent surveillance systems, and robot technology [1,2,3,4]. Traditional action recognition methods use RGB image, video data, and depth image data [5,6,7,8]. Compared with these data, the skeleton data contain three-dimensional spatial and temporal information. Skeleton data are less affected by external factors and have high robustness and computational efficiency. Therefore, skeleton-based action recognition has received extensive attention in recent years.

Early skeleton-based action recognition methods usually analyzed skeleton spatial patterns through handcrafted methods [9,10,11,12]. With the continuous development of deep learning, many methods extract motion patterns to form a skeleton sequence and use RNNs to model them [13,14,15,16,17]. Other methods based on CNNs transform skeleton data into pseudo images and send them into CNNs for prediction [18,19,20,21,22,23]. However, these methods cannot capture the inherent spatial relationship between joints.

Graph convolution neural networks [24,25,26,27,28,29,30] have made significant progress and have been successfully applied in many fields in recent years. Different from traditional CNN and RNN models, which can only be used for grid-based data, graph convolution neural networks comb data with a generalized topology and deeply study its characteristics and rules. The human skeleton is a graph with a stable topological structure. Yan et al. [31] first proposed spatial temporal graph convolutional networks (ST-GCNs) in skeleton-based action recognition and showed impressive improvements. The spatiotemporal skeleton graph in ST-GCNs adds spatial connections to any adjacent joints of the natural physical structure of the skeleton and adds temporal connections between consecutive frames of the same joint. The spatial domain information and temporal domain information of skeleton data are learned by spatial graph convolution and temporal convolution, respectively. The spatiotemporal graph structure in ST-GCNs is defined by the physical structure of the human skeleton, which leads to the model focusing on the vertexes of adjacent joints in the physical structure and ignoring the joints’ vertexes, which are closely related in movement, but far apart in physical structure. Therefore, many methods have been put forward to solve this problem recently. Shi et al. [32] proposed the 2s-AGCNmodel, which adds an adaptive graph and a graph structure similar to the global self-attention mechanism so that joints’ vertexes far apart in physical structure can also transmit information. Li et al. [33] proposed structural links for higher order relationships between joints and actional links to learn action-specific dependencies.

**Figure 1:**
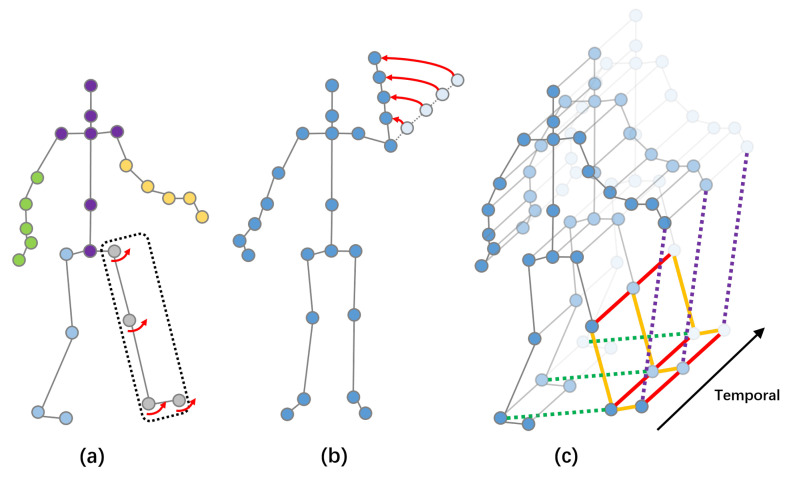
(**a**) An example of similar movement trends of the same part. (**b**) An example of joints near the edge moving more significantly than joints near the center. (**c**) The spatial temporal graph of a skeleton sequence, in which the red lines connect two joints with temporal correlation, orange lines connect two joints with spatial correlation, green lines connect two joints with symmetric correlation, and purple lines connect two joints with edge correlation.

Nevertheless, these methods still ignore some issues: (1) When the human completes an action, the movement characteristics of various parts of the body vary greatly. For example, as shown in Figure 1a, during walking, the right and left shoulders are very close in physical structure, but their movement direction is almost the opposite, while the movement characteristics of feet and legs in the same part tend to be consistent. Therefore, a simple graph convolution of them as a whole may weaken some of the more significant motion features of the limbs or parts. (2) In the process of completing an action, there is fixed coordination and relevance between many joints. For example, the left hand and right hand would swing involuntarily at the same time during walking. (3) The movement characteristics of some edge joints are more significant than those of joints near the center in movement. For example, as shown in Figure 1b, in the process of waving, the movement characteristics of the tip of the hand are more significant than that of the arm, but these joints will be treated equivalently in the ordinary graph convolution process.

**Figure 2:**
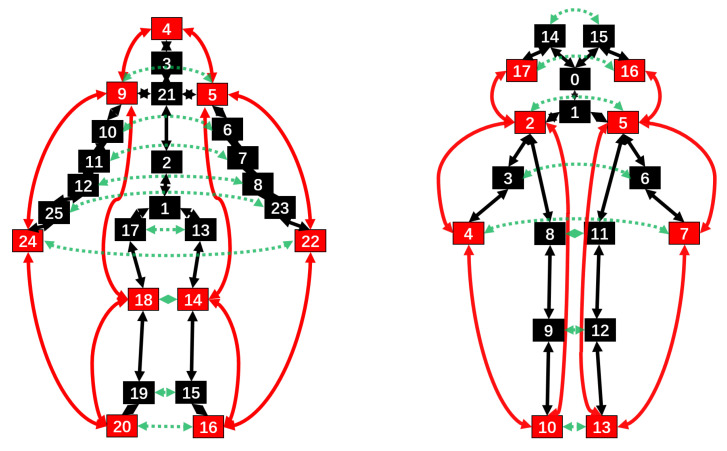
The connection structure of the skeleton graphs in the whole graph convolution network (WGCN). The left graph represents the skeleton graph of the NTU RGB+D 60 and NTU RGB+D 120 datasets, and the right graph represents the skeleton graph of the Kinetics Skeleton dataset. The number on the joints represents the joints labels. The black joints represent the ordinary joints; the red joints represent the edge joints; the black connection between the two joints’ vertexes represents the physical structure connection; the red connection between the two edge joints represents the edge connection; the green connection between the two symmetric joints represents the symmetric connection.

In this work, we propose two new graph convolution methods to address the above issues: the whole graph convolution network (WGCN) and the part graph convolution network (PGCN). In WGCN, we construct two new connection methods according to the movement patterns of humans: symmetric connection and edge connection. Symmetrical connection and edge connection are shown in Figure 2. Symmetric connection captures the potential dependence of symmetric joints’ vertexes with coordination, while edge connection captures the movement characteristics of some edge joints’ vertexes with more significant movement characteristics. They combine with the physical structure connection of the human skeleton to learn the whole scale skeleton spatial features and finally add temporal convolution to learn the whole scale skeleton spatiotemporal features in skeleton data. WGCN is built and improved based on the graph convolution unit of AGCN [32]. In PGCN, according to the different parts of the human body structure (left arm, right arm, left leg, right leg, torso), the skeleton graph of the human body is divided into five subgraphs. The five subgraphs are separately convoluted and combined with temporal convolution to learn the spatiotemporal features of each part. PGCN was inspired by some early RNN-based and CNN-based methods [13,17,23] that divide skeleton data into multiple temporal sequences of the human skeleton parts. We construct a whole and part adaptive fusion graph convolutional neural network, which contains two branch networks. Multiple WGCN units stack as the leading branch network to extract whole skeleton features, and multiple PGCN units stack as the auxiliary branch network to extract unique part scale skeleton features. Moreover, an adaptive fusion module is proposed to fuse the two kinds of features adaptively. The two features go through adaptive fusion modules several times, resulting in richer and more effective skeleton features for action recognition.

Experiments were conducted to verify the effectiveness of our model on three large-scale datasets of skeleton-based action recognition: NTU RGB+D 60 [34], NTU RGB+D 120 [35], and Kinetics Skeleton 400 [36]. Our model achieves state-of-the-art performance on the three datasets. We also conducted ablation experiments on each component of the model to verify the importance of each component.

The main contributions of this work are listed as follows:

1. We propose two new graph convolution methods for skeleton-based action recognition based on the movement patterns of human action: whole graph convolution (WGCN) and part graph convolution (PGCN), which are used to learn the whole scale skeleton feature and the part scale skeleton feature, respectively.
2. We propose an adaptive fusion module, which takes advantage of the complementarity of the whole scale skeleton feature and the part scale skeleton feature to make the two kinds of features adaptively fuse to obtain a more abundant and effective skeleton feature.
3. We build a whole and part adaptive fusion graph convolutional neural network (WPGCN), and its performance of exceeds the state-of-the-art methods on three large-scale datasets for skeleton-based action recognition.

## 2. Related Works

In this section, we briefly review the relevant research literature from the following aspects: non-GCN-based methods for skeleton-based action recognition, GCN-based methods for skeleton-based action recognition, and methods based on the part feature for skeleton-based action recognition.

### 2.1. Non-GCN-Based Methods

Conventional skeleton-based action recognition methods always use handcrafted methods [9,10,11,12] to analyze spatial patterns. It is difficult to extend the designed model to other applications, and they have limited performance.

The CNN-based methods transform 3D-skeleton sequence data from a vector sequence to a pseudo-image and use the CNN network to extract the pseudo-image features [18,19,20,21,22,23]. The advantage of the CNN method lies in its powerful feature extraction capabilities, but it cannot handle the spatial and temporal relationships in skeleton information well.

The RNN-based methods [13,14,15,16,17] regard 3D-skeleton sequence data as a temporal sequence of vectors and feed a sequence of skeletons directly into several recurrent neural networks. The RNN method itself can model the temporal information, but due to the weakness of the spatial modeling ability of the RNN-based architecture, the performance of some related methods generally could not gain a competitive result.

These methods have obvious limitations because it is difficult to capture the spatial information of the human skeleton data.

### 2.2. GCN-Based Methods

The GCN-based methods have received widespread attention due to their high efficiency. Compared with CNN-based and RNN-based methods, GCN can model the spatial features of the human skeleton. Yan et al. [31] first proposed a spatial and temporal graph convolutional network ST-GCN, which uses spatial graph convolution and temporal convolution for spatial-temporal modeling. Shi et al. [32] proposed the 2s-AGCN model, which constructs an adaptive graph to give adaptive attention to each joint. The model also takes the original joint 3D coordinates as the joint stream data and takes the 3D coordinate difference of two adjacent joints as the bone stream data to construct a two-stream structure framework of the bone stream and joint stream. Shi et al. [37] continued to add attention mechanisms and a multi-stream structure to 2s-AGCN and proposed a new improved model MS-AAGCN. Li et al. [33] proposed actional links to learn action-specific dependencies and structural-links (S-links) for higher order relationships between joints. Shi et al. [38] represented the skeleton data as a directed acyclic graph to model the dependencies between joints and bones and designed a directed graph neural network to extract dependencies for the action recognition task. Si et al. [39] fused graph convolution with LSTM to construct a fusion network AGC-LSTM, which uses graph convolution to extract spatial information and use LSTM to extract temporal information. Gao et al. [40] designed BAGCN, which learns spatial-temporal context from human skeleton sequences leveraging a graph convolutional neural network-based focusing and diffusion mechanism. Liu et al. [41] introduced a convolution operator of a cross-spatiotemporal aggregation graph to enhance the cross-spatiotemporal aggregation ability of skeleton information. These methods use graph convolution to extract the spatiotemporal features of the human skeleton. Based on these methods, we constructed two graph convolution methods to extract the skeleton features of the whole scale and the skeleton features of the part scale. By fusing the two skeleton features, we get more abundant skeleton features for the final action recognition.

### 2.3. Methods Based on the Part Feature

Du et al. [13] first divided the human skeleton into five parts and then constructed a recurrent neural network to model it. The five parts of the skeleton were fed to subnetworks. Si et al. [17] extracted the spatial features of the skeleton from different parts separately. Minh et al. [23] constructed a CNN network to capture fine-grained features in various parts of the human skeleton. Although these methods capture the unique characteristics of each part, they ignore the correlation within the movement patterns of different parts and the complementarity of the whole feature to the part feature. It is not complete to use only the features separated from each part in the spatial domain, because these local features still have some connections in the spatial domain, such as the connectivity between the left hand and the trunk and the symmetry of the left leg and the right leg. We were inspired by these methods to construct PGCN to extract the unique features of each part to supplement the whole scale skeleton features.

## 3. Methods

In this section, we first review the background of spatiotemporal graph convolution in skeleton-based action recognition. Then, we would elaborate on the implementation details of our network.

### 3.1. Background

The skeleton data of human action recognition are composed of a series of temporal sequences, including the three-dimensional coordinates of human joints in the time of completing the action. A skeleton sequence of C×T×N with C channels, N joints, and T frames is represented as an undirected spatiotemporal graph G = {V, E}, where V = {$v_{n}^{t}|n=1,2,\ldots ,N;t=1,2,\ldots ,T$} represents the set of all joints’ vertexes and E denotes the edge set between joints’ vertexes determined by the adjacent matrix $\bar{A}$ in the spatial domain and determined by the connection between consecutive frames in the temporal domain. The adjacent matrix $\bar{A}$∈${\left\{0,1\right\}}^{N\times N}$ is defined according to the spatial structure of the vertexes. Specifically, if vertex $v_{i}$ and vertex $v_{j}$ are physically adjacent, ${\bar{A}}_{ij}$ = 1; otherwise, ${\bar{A}}_{ij}$ = 0. $\bar{A}$ needs to be normalized by using its degree matrix as:

(1) $$A_{norm}=\Lambda^{-\frac{1}{2}}\bar{A}\Lambda^{\frac{1}{2}}$$

Λ is the normalized diagonal matrix of $\bar{A}$, and its elements can be expressed as $\Lambda_{ii}=\sum_{j=0}^{N}{\bar{A}}_{ij}+\epsilon$. We follow the recent method [32] and set ϵ to 0.001 to prevent invalid calculations.

In the temporal domain, a temporal convolution is selected to extract the temporal information of skeleton data.

For a skeleton feature X of layer l, the updating rules of completing a graph convolution transformation in the spatial domain can be expressed as Equation (2):

(2) $$X^{\left(l+1\right)}\left(v_{i}\right)=\sum_{j=1}^{N}\Lambda^{-\frac{1}{2}}{\bar{A}}_{ij}\Lambda^{\frac{1}{2}}X^{\left(l\right)}\left(v_{j}\right)W^{\left(l\right)}$$

where N represents the total number of joints’ vertexes, $X^{\left(l\right)}$ is the input skeleton feature, $X^{\left(l+1\right)}$ is the output skeleton feature, W denotes a $C_{in}^{\left(l\right)}\times C_{out}^{\left(l\right)}\times 1\times 1$ weight vector of the convolution kernel size of 1×1. $C_{in}^{\left(l\right)}$, and $C_{out}^{\left(l\right)}$ denotes the number of input and output channels of the skeleton features of layer l.

The updating rules for completing a spatiotemporal graph convolution transformation can be summarized as Equation (3):

(3) $$X^{\left(l+1\right)}\left(v_{i}\right)=F_{t}\left(\sigma \left(\sum_{j=1}^{N}\Lambda^{-\frac{1}{2}}{\bar{A}}_{ij}\Lambda^{\frac{1}{2}}X^{\left(l\right)}W^{\left(l\right)}\right)\right)$$

where $F_{t}$ is the temporal convolution layer and σ is an activation function.

### 3.2. Whole Graph Convolutional Network Unit

The function of the whole graph convolutional network (WGCN) is to take all the joints’ vertexes of the human skeleton as a whole to perform graph convolution so that the skeleton information can be spread efficiently, so as to obtain the whole scale skeleton spatiotemporal feature.

The traditional skeleton graph structure is based on the physical structure of the human skeleton, which is obviously inefficient for data transmission. It also ignores the movement patterns of the human during the action. In order to transfer skeleton information more effectively in WGCN, we add two kinds of new relationship connections (the symmetric connection and the edge connection) on the existing graph convolution based on human physical structure dependence according to human movement patterns.

Specifically, the graph convolution operator of WGCN is composed of four kinds of relation connections modes: self-loop connection, physical structure connection, symmetric connection, and edge connection. These four connections are captured by four vertex connection matrices. We define these four connection matrices as: ${\bar{A}}_{loop}$, ${\bar{A}}_{physical}$, ${\bar{A}}_{symmet}$, and ${\bar{A}}_{edge}$.

The self-loop connection and ${\bar{A}}_{loop}$: ${\bar{A}}_{loop}$ is an N × N identity matrix. The self-loop connection allows joints’ vertexes to retain part of their data information during the graph convolution process, ensuring the stability of the information of each node in the graph convolution process. ${\bar{A}}_{loop}$ can be expressed as follows:

(4) $${\bar{A}}_{loop}=I^{N\times N}$$

The physical structure connection and ${\bar{A}}_{physical}$: The ${\bar{A}}_{physcial}$ is the same as $\bar{A}$ in Equation (2). It represents the N × N adjacent matrix of the physical structure of the human skeleton. The physical structure connection enables skeleton information to be transmitted with the physical connection of joints’ vertexes, thus ensuring the physical dependence between adjacent joints during the movement. ${\bar{A}}_{physical}$ can be expressed as Equation (5):

(5) $${\bar{A}}_{physical(ij)}=\left\{\begin{array}{cc} 1 & \mathrm{vertexes}\;v_{i}\;\mathrm{and}\;v_{j}\;\mathrm{are}\;\mathrm{adjacent}\\ 0\; & \mathrm{otherwise} \end{array}\right.$$

The symmetric connection and ${\bar{A}}_{symmet}$: Considering the symmetric structure of the human body, as well as the strong correlation and coordination of various joint parts in the process of human movement, this correlation and coordination are most obvious in the symmetric joints. For example, in the process of walking, people’s left hand and right hand would regularly swing at the same time, and the left shoulder and right shoulder would show almost the same frequency of movement. Therefore, we use a new symmetric connection to capture this coordination and correlation. The symmetric connection can also improve the efficiency of skeleton data for graph convolution in the whole skeleton. We define the N × N connection matrix ${\bar{A}}_{symmet}$ as Equation (6):

(6) $${\bar{A}}_{symmet(ij)}=\left\{\begin{array}{cc} 1 & \mathrm{vertexes}\;v_{i}\;\mathrm{and}\;v_{j}\;\mathrm{are}\;\mathrm{symmetric}\\ 0 & \mathrm{otherwise} \end{array}\right.$$

The edge connection and ${\bar{A}}_{edge}$: Consider that in the process of human movement, the movement characteristics of some edge joints are more obvious than those of the joints closer to the center; for example, in the process of waving, the movement characteristics of the hand are more significant than those of the arms. Obviously, these key joints’ vertexes have more important movement information. However, in the graph convolution only based on physical structure connection, these key joints’ vertexes are in the edge position in the physical structure, and they have only one adjacent joint vertex. In the dissemination of movement information, they can only disseminate information to one joint vertex, which results in a great limitation of data information dissemination. Therefore, we add an edge connection in the graph convolution network. As shown in the red vertexes in Figure 2, the edge joints we define consist of two types of joints: one is that there is only one adjacent joint vertex in the physical structure, such as the left hand, the right hand, the left toe, the right toe, and the head; the other is some joints’ vertexes that play an important role during the movement as the real edge joint vertexes, such as the left knee, right knee, left shoulder, and right shoulder. We add the second kind of pseudo edge joints to the edge joints mainly to enhance the importance of these joints in graph convolution, rather than making them equal to ordinary joints. We create a new edge connection between these edge vertexes as shown by the red connection between two vertexes in Figure 2, which allows remote edge vertexes to transmit data. This connection increases the importance of edge joints’ vertexes and the efficiency of graph convolution of skeleton data over the whole skeleton. We define the N × N connection matrix ${\bar{A}}_{edge}$ as Equation (7):

(7) $${\bar{A}}_{edge(ij)}=\left\{\begin{array}{cc} 1 & \mathrm{vertex}\;v_{i}\;\mathrm{has}\;\mathrm{an}\;\mathrm{edge}\;\mathrm{connection}\;\mathrm{with}\;v_{j}\\ 0 & \mathrm{otherwise} \end{array}\right.$$

${\bar{A}}_{loop}$, ${\bar{A}}_{physical}$, ${\bar{A}}_{symmet}$, and ${\bar{A}}_{edge}$ need to be normalized by the corresponding degree matrix like Equation (1). We can get the normalized relation matrix $A_{loop}$, $A_{physical}$, $A_{symmet}$, $A_{edge}$:

(8) $$A_{loop}={\bar{A}}_{loop}$$

(9) $$A_{physical}=\Lambda_{p}^{-\frac{1}{2}}{\bar{A}}_{physical}\Lambda_{p}^{\frac{1}{2}}$$

(10) $$A_{symmet}=\Lambda_{s}^{-\frac{1}{2}}{\bar{A}}_{symmet}\Lambda_{s}^{\frac{1}{2}}$$

(11) $$A_{edge}=\Lambda_{e}^{-\frac{1}{2}}{\bar{A}}_{edge}\Lambda_{e}^{\frac{1}{2}}$$

In detail, according to Equation (2), the updating rules of WGCN in the spatial domain can be described as Equation (12):

(12) $$\begin{array}{c} X^{\left(l+1\right)}\left(v_{i}\right)=\sum_{j=1}^{N}(A_{loop(ij)}X^{\left(l\right)}\left(v_{j}\right)W_{l}^{\left(l\right)}+A_{physical(ij)}X^{\left(l\right)}\left(v_{j}\right)W_{p}^{\left(l\right)}+A_{symmet(ij)}X^{\left(l\right)}\left(v_{j}\right)W_{s}^{\left(l\right)}\\ +A_{edge(ij)}X^{\left(l\right)}\left(v_{j}\right)W_{e}^{\left(l\right)}+C_{k(ij)}X^{\left(l\right)}\left(v_{j}\right)W_{c}^{\left(l\right)}) \end{array}$$

Since the weights are not shared, $W_{l}$, $W_{p}$, $W_{s}$, $W_{e}$, and $W_{c}$ are used to represent five different $C_{in}\times C_{out}\times 1\times 1$ weight vectors of the convolution kernel size of 1×1. We set $A_{loop}$, $A_{physical}$, $A_{symmet}$, and $A_{edge}$ as learnable matrices, and their elements can be optimized together with other parameters in the network, which increases the flexibility of the network. The matrix $C_{k}$ in Equation (12) is an attention matrix of the self-attention mechanism, which is used to capture the correlation strength between vertexes in each skeleton feature.

**Figure 3:**
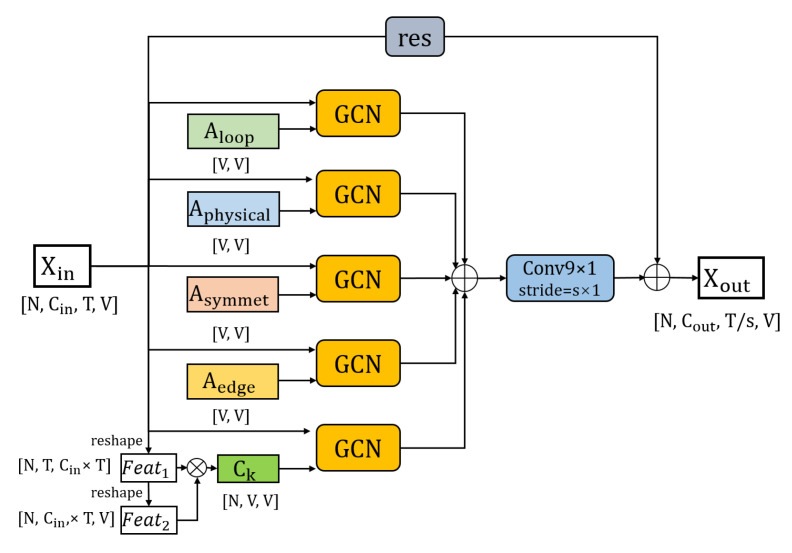
The structure of the WGCN unit. ⊕ denotes the element-wise summation.

The structure of the WGCN unit is shown in Figure 3. The input skeleton feature $X_{in}$∈$R^{C_{in}\times T\times N}$ is graph convoluted with the graph convolution operators $A_{loop}$, $A_{physical}$, $A_{symmet}$, $A_{edge}$, and $C_{k}$ to get the new skeleton features, respectively. Then, these features are element-wise added to get the skeleton feature of the middle layer $X_{mid}$∈$R^{C_{out}\times T\times N}$. A temporal convolution with a kernel size of 9×1 is used to extract temporal domain information from the skeleton data. The temporal convolution has different strides (set to 1 × 1 or 2 × 1) in different WGCN units. We add a residual connection structure to each WGCN unit. In the residual connection, if the number of the channels of the input feature and the output feature in the WGCN unit is the same, the input feature is directly added to the output feature. If the channel number of the input feature and the output feature in the WGCN unit is different, we use an average pooling layer whose kernel size and stride are 2×1 to adjust the size of the input feature time frame, then use a convolution layer with a kernel size of 1×1 to adjust the number of channels, and then add it to the output feature. The final output feature of WGCN is $X_{out}$∈$R^{C_{out}\times T/s\times N}$, where s is the time domain dimension component of the stride of the temporal convolution.

### 3.3. Part Graph Convolutional Network Unit

The motivation for introducing the part graph convolutional network (PGCN) is to be able to extract skeleton features from the part scale as a supplement to WGCN. Specifically, when a human completes an action, the movement characteristics of the joints on different limbs of the human body are very different. For example, the movement trends of hands and feet are very different when walking, but the movement patterns of hands and arms are very similar. However, for a method similar to WGCN, all parts of the human body are regarded as a whole for graph convolution, and the deep skeleton features of each joints’ vertexes obtained by multi-layer graph convolution will be gradually assimilated, leading to the weakening of the feature difference of each vertex. This may weaken the significant features of some important parts, thus limiting the effect of action recognition. Therefore, we construct the part graph convolutional network to extract the unique part scale skeleton features.

**Figure 4:**
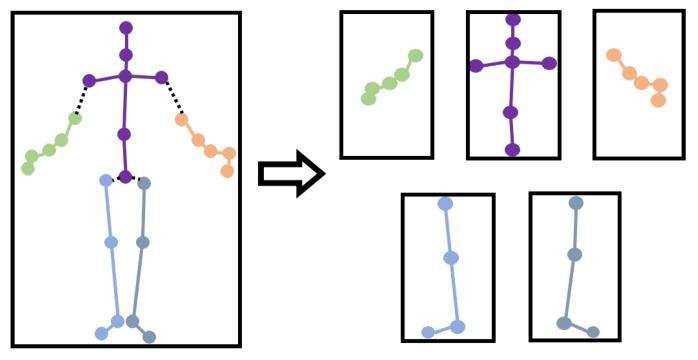
The division of subgraphs. The left figure shows the whole skeleton graph, and the right figure shows five subgraphs, which represent the left arm, torso, right arm, left leg, and right leg, respectively.

According to the structure of the human body, we divide the whole human body into five parts (left arm, right arm, left leg, right leg, and trunk). As shown in Figure 4, the skeleton graph of the human body is divided into five corresponding subgraphs. Then, PGCN is used to graph convolute the five skeleton subgraphs to obtain the unique deep skeleton features of each part of the human skeleton.

In PGCN, we define three connection methods of graphs: self-loop connection, part outward connection, and part inward connection. Their corresponding graph convolution connection matrices are ${\bar{A}}_{loop}$, ${\bar{A}}_{pin}$ and ${\bar{A}}_{pout}$.

The self-loop connection and ${\bar{A}}_{loop}$ are consistent with those in WGCN.

**Figure 5:**
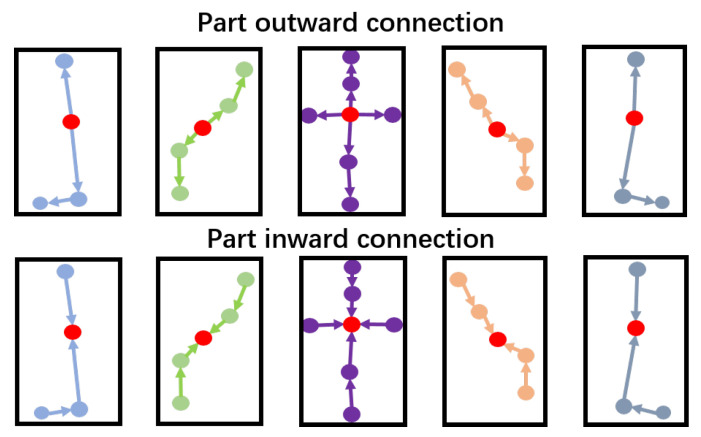
The part outward connection and the part inward connection structure in PGCN. The red joints’ vertexes represent the center joints’ vertexes of each part. The arrow direction indicates the update direction from the source vertex to the target vertex.

The part outward connection and the part inward connection: As shown in Figure 5, we select a center joint vertex (the red joint vertex in Figure 5 in each part as the center joint vertex of each part graph. In the part outward connection, for any two adjacent vertexes in each part, we take the vertex closest to the center vertex or the center vertex as the source vertex and the vertex far away from the center vertex as the target vertex. Similarly, in the part inward connection, for any two adjacent vertexes in each part, we take the vertex closest to the center vertex or the center vertex as the target vertex and the vertex far away from the center vertex as the source vertex. Each graph convolution updates the vertex attributes from the source vertex to the target vertex. ${\bar{A}}_{pin}$ and ${\bar{A}}_{pout}$ can be expressed as Equations (13) and (14):

(13) $${\bar{A}}_{pin}=\left\{\begin{array}{cc} 1 & \mathrm{if}\;v_{j}\;\mathrm{is}\;\mathrm{the}\;\mathrm{inward}\;\mathrm{target}\;\mathrm{vertex}\;\mathrm{of}\;v_{i}\;\\ \alpha & \mathrm{else}\;\mathrm{if}\;v_{i}\;\mathrm{and}\;v_{j}\;\mathrm{are}\;\mathrm{located}\;\mathrm{in}\;\mathrm{the}\;\mathrm{same}\;\mathrm{part}\\ 0 & \mathrm{otherwise} \end{array}\right.$$

(14) $${\bar{A}}_{pout}=\left\{\begin{array}{cc} 1 & \mathrm{if}\;v_{j}\;\mathrm{is}\;\mathrm{the}\;\mathrm{outward}\;\mathrm{target}\;\mathrm{vertex}\;\mathrm{of}\;v_{i}\;\\ \alpha & \mathrm{else}\;\mathrm{if}\;v_{i}\;\mathrm{and}\;v_{j}\;\mathrm{are}\;\mathrm{located}\;\mathrm{in}\;\mathrm{the}\;\mathrm{same}\;\mathrm{part}\\ 0 & \mathrm{otherwise} \end{array}\right.$$

We set a tiny parameter α = 0.001 for any two vertexes that are in the same part that are not the source vertex and the target vertex to enhance the connectivity of each vertex in the same part. ${\bar{A}}_{loop}$, ${\bar{A}}_{pin}$, and ${\bar{A}}_{pout}$ need to be normalized by the corresponding degree matrix like Equation (1). We can get the normalized relation matrix $A_{loop}$, $A_{pin}$,$A_{pout}$:

(15) $$A_{loop}={\bar{A}}_{loop}$$

(16) $$A_{pin}=\Lambda_{pin}^{-\frac{1}{2}}{\bar{A}}_{pin}\Lambda_{pin}^{\frac{1}{2}}$$

(17) $$A_{pout}=\Lambda_{pout}^{-\frac{1}{2}}{\bar{A}}_{pout}\Lambda_{pout}^{\frac{1}{2}}$$

Suppose the elements of $A_{loop}$, $A_{pin}$, and $A_{pout}$ are set as learnable parameters like WGCN. In that case, it may cause the mutual transmission of feature information between joints in different parts, and the purpose of retaining the uniqueness of features of each part cannot be achieved. Therefore, we set up three N × N learnable weight matrices $M_{l}$, $M_{i}$, and $M_{o}$ to learn the strength of joint information transmission within each part. In detail, according to Equation (2), the updating rules of PGCN in the spatial domain can be described as Equation (18):

(18) $$\begin{array}{c} X^{\left(l+1\right)}\left(v_{i}\right)=\sum_{j=1}^{N}[\left(M_{l(ij)}⊗A_{loop(ij)}\right)X^{\left(l\right)}\left(v_{j}\right)W_{lp}^{\left(l\right)}+\left(M_{i(ij)}⊗A_{pin(ij)}\right)X^{\left(l\right)}\left(v_{j}\right)W_{i}^{\left(l\right)}\\ +\left(M_{o(ij)}⊗A_{pout(ij)}\right)X^{\left(l\right)}\left(v_{j}\right)W_{o}^{\left(l\right)}] \end{array}$$

Since the weights are not shared, $W_{lp}$, $W_{i}$, and $W_{o}$ are used to represent three different $C_{in}\times C_{out}\times 1\times 1$ weight vectors of the convolution kernel size of 1×1. Besides, we define three N × N learnable weight matrices $M_{l}$, $M_{i}$, and $M_{o}$ as follows: if vertex $v_{i}$ and vertex $v_{j}$ are in the same part, then the element $M_{ij}$ is set as a learnable parameter, and the initial value is set to 1; otherwise the element $M_{ij}$ is set to constant 0. ⊗ denotes element-wise multiplication.

**Figure 6:**
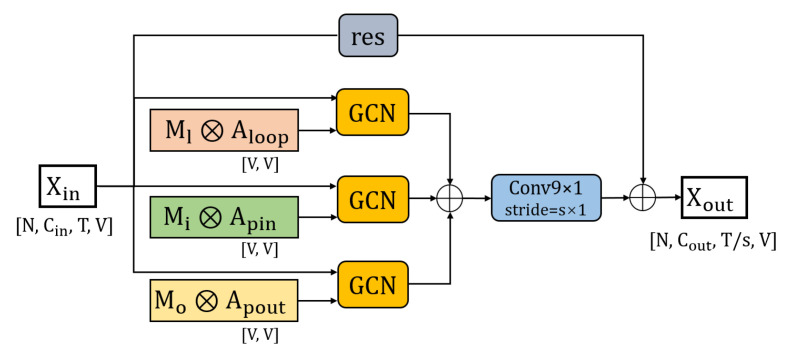
The structure of the PGCN unit. ⨁ denotes the element-wise summation. ⨂ represents the element-wise multiplication.

The structure of the PGCN unit is shown in Figure 6. The three graph convolution operators $A_{loop}$, $A_{pin}$, and $A_{pout}$ are multiplied by their corresponding weight matrix $M_{l}$, $M_{i}$, and $M_{o}$ before the graph convolution. The input skeleton feature $X_{in}$∈$R^{C_{in}\times T\times N}$ is graph convoluted with the graph convolution operators multiplied by weight M to get the new skeleton features. Then, these features are element-wise added to get the skeleton feature of the middle layer $X_{mid}$∈$R^{C_{out}\times T\times N}$. A temporal convolution with a kernel size of 9×1 is used to extract temporal domain information from skeleton data. Different strides (1 × 1 or 2 × 1) are selected for the temporal convolution in different PGCN units. We also add a residual connection for each PGCN unit that is consistent with WGCN. The final output feature of WGCN is $X_{out}$∈$R^{C_{out}\times T/s\times N}$.

### 3.4. Whole and Part Adaptive Fusion Graph Convolutional Network

#### 3.4.1. Adaptive Fusion Module

**Figure 7:**
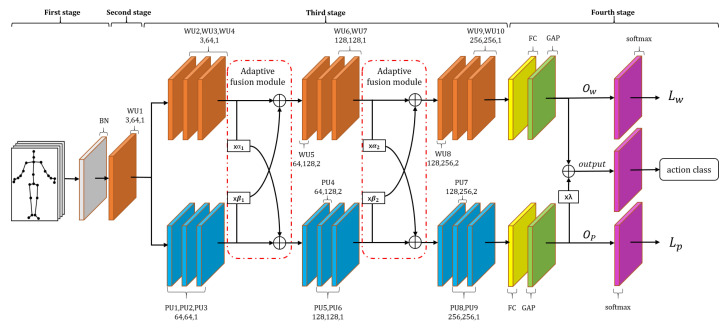
The overall architecture of the whole and part adaptive fusion graph convolutional neural network (WPGCN). There is a total of 10 WGCN units (WU1-WU10) and nine PGCN units (PU1-PU9). The three numbers of each unit represent the number of input channels, the number of output channels, and the stride, respectively. $\alpha_{1}$, $\alpha_{2}$, $\beta_{1}$, $\beta_{2}$, and λ denotes the fusion parameters. ⨁ denotes the element-wise summation.

As we explained above, the deep whole scale skeleton feature and the deep part scale skeleton feature can be learned from WGCN and PGCN. In order to make full use of the complementarity and the difference between the two kinds of features, we propose a feature adaptive fusion strategy. As shown in Figure 7, we use $X_{w}$ and $X_{p}$ to represent the whole scale skeleton feature and the part scale skeleton feature from the WGCN unit and PGCN unit in turn. Then, we make a complementary adaptive fusion transformation of $X_{w}$ and $X_{p}$:

(19) $$Y_{p}=X_{p}+\alpha X_{w}$$

(20) $$Y_{w}=X_{w}+\beta X_{p}$$

where α and β are the fusion parameters of the two features. It is worth noting that we set α and β as learnable parameters. They can be trained together with other parameters in the network, and the initial value is set to 0. Through this data-driven method, the fusion features we get are more adaptive and flexible.

#### 3.4.2. Overall Architecture

The final model architecture is illustrated in Figure 7. The overall architecture of our network can be divided into four stages.

In the first stage, the input skeleton sequence passes through a data batch normalization layer for normalization processing.

In the second stage, the feature first passes through the WGCN unit, and the number of skeleton feature channels changes from three to 64.

In the third stage, the skeleton feature is divided into two branches. One branch path passes through the stacked 9 WGCN units to get the deep whole scale skeleton features, and the other branch passes through 9 PGCN units to get the deep part scale skeleton features. The output channels of these WGCN units and PGCN units are 64, 64, 64, 128, 128, 128, 256, 256, and 256 in order. The corresponding temporal convolution strides are 1 × 1, 1 × 1, 1 × 1, 2 × 1, 1 × 1, 1 × 1, 1 × 1, 2 × 1, 1 × 1, 1 × 1, and 1 × 1, respectively. We set up two adaptive feature fusion modules after the third and sixth WGCN unit and PGCN unit to increase the richness of the two features.

In the fourth stage, we send the extracted whole scale skeleton features and part scale skeleton features to the global average pooling layer and fully connected layer, and we will get two outputs $O_{w}$ and $O_{p}$ after the FC layer. Our final output is obtained by fusing $O_{w}$ and $O_{p}$, and the fusion parameter is λ:

(21) $$output=O_{w}+\lambda O_{p}$$

This output is used by the softmax classifier to get a vector predicting each action probability for the final action recognition.

We take the WGCN unit of the second stage, the 9 WGCN units of the third stage, and the corresponding FC layer and global average pooling layer as the leading branch network. We regard the 9 PGCN units in the third stage and the corresponding FC layer and global average pooling layer as the auxiliary branch network.

#### 3.4.3. Loss of Network

The output $O_{w}$ and $O_{p}$ in Equation (21) are the predicted action classes confidence scores. These scores can be normalized by the softmax classifier to get vectors predicting each action. $p_{w}$ and $p_{p}$ represent the output of the softmax operator, then the probability of class i-th action $p_{w(i)}$, $p_{p(i)}$ can be calculated as Equations (22) and (23):

(22) $$p_{w(i)}=\frac{\exp\left(O_{w(i)}\right)}{\sum_{j=1}^{k}\exp\left(O_{w(j)}\right)}$$

(23) $$p_{p(i)}=\frac{\exp\left(O_{p(i)}\right)}{\sum_{j=1}^{k}\exp\left(O_{p(j)}\right)}$$

In the training process, we optimize it as a dual-task learning problem with cross-entropy loss. The loss components $L_{w}$ and $L_{p}$ of the two classifiers are calculated as Equations (24) and (25):

(24) $$L_{w}\left(y,p_{w}\right)=-\sum_{i=1}^{k}y_{i}\log\left(p_{w(i)}\right)$$

(25) $$L_{p}\left(y,p_{p}\right)=-\sum_{i=1}^{k}y_{i}\log\left(p_{p(i)}\right)$$

where k is the total number of action classes and y is the action label; if y = i, $y_{i}$ is set as 1; otherwise, it is set as 0. The final loss of the model can be summarized as Equation (26):

(26) $$L=L_{w}+\lambda L_{p}$$

λ is the fusion parameter, which is the same as that of Equation (21).

#### 3.4.4. Two-Stream Framework

Bone has proven to be another form of spatial information as important as joints and has been used in many recent methods [32,37,38,41,42,43,44]. The original skeleton data only contain the 3D coordinates of all joints in the skeleton, and the bone stream data are obtained by vector calculation of the original joint stream data. Specifically, each three-dimensional bone vector is the vector difference between two adjacent joints. For example, for the two adjacent joint vertices $v_{1}$ = $\left(x_{1},y_{1},z_{1}\right)$, $v_{2}$ = $\left(x_{2},y_{2},z_{2}\right)$, the bone composed of these two joint vertices can be expressed as $e_{v_{1},v_{2}}$ = $\left(x_{2}-x_{1},y_{2}-y_{1},z_{2}-z_{1}\right)$. The number of bones is one less than the number of joints, and an empty bone with a value of 0 is added to the center joint to make sure that each bone can correspond to a unique joint. Joint stream data and bone stream data are sent to two identical WPGCN models, and they are trained independently. In the test process, both streams produce the prediction scores. The softmax scores from the joint stream and bone stream are added to generate the final prediction score.

## 4. Experiments

In this section, we evaluate our model on three large-scale skeleton action recognition datasets, NTU RGB+D 60 [34], NTU RGB+D 120 [35], and Kinetics Skeleton [36], and compare it with some of the state-of-the-art methods to prove the effectiveness of our model. In addition, we add ablation experiments to demonstrate the importance of each component in the model.

### 4.1. Datasets

#### 4.1.1. NTU RGB+D 60

NTU RGB+D 60 [34] is currently one of the largest and most widely used skeleton-based action recognition datasets. The dataset contains more than 56,000 skeleton sequences, divided into 60 action classes. Forty volunteers performed these skeleton sequence samples. Only one or two volunteers appear in each skeleton sequence, and each person in each frame contains three-dimensional coordinates of 25 main body joints. We evaluated based on two benchmarks proposed by the author: cross-subject (X-Sub) and cross-view (X-View). In cross-subject, the skeleton sequences of 20 subjects are used as the training set, and the skeleton sequences of the remaining 20 subjects are used as the test set. The training set and the test set have 40,320 and 16,560 sequences, respectively. In the cross-view, the data training set and the test set are divided according to the camera view angle. The training set contains 37,920 skeleton sequences captured from the front and two side views, while the test set has 18,960 skeleton sequences captured from the left and right 45 degree views.

#### 4.1.2. NTU RGB+D 120

NTU-RGB-D 120 [35] is an extended dataset of NTU RGB+D 60, with 114,480 skeleton sequence samples and 120 action classes. There were 106 subjects and 32 setup IDs in the dataset. Similar to NTU RGB+D 60, the author defines two recommended benchmark assessments: cross-subject (X-Sub) and cross-setup (X-Set). In the cross-subject, the training set is composed of the skeleton sequences of 53 subjects, and the test set is composed of the skeleton sequences of the other 53 subjects. In the cross-setup, the 16 IDs sequences constitute the training set, and the remaining 16 IDs sequences constitute the test set.

#### 4.1.3. Kinetics Skeleton 400

Kinetics Skeleton 400 [36] is a large dataset for human action recognition, including 300,000 video clips from YouTube and 400 action classes. It only contains raw video clip samples without skeleton sequences, and each original video clip lasts about 10 s. The public available OpenPose [45] toolbox is used to estimate the position of 18 joints in each frame of the clip. Based on the average joint confidence, two people were selected for multi-person video editing. We used their published data (dynamics skeleton) to evaluate our model. We evaluated the recognition performance of our model according to the classification accuracy of the top 1 and top 5 recommended by the dataset author. The dataset provides a training set of 240,000 clips and a test set of 20,000 clips.

### 4.2. Training Details

All of our experiments were implemented on the PyTorch deep learning framework, and our model was trained on 4 GTX-1080Ti GPUs. In the data processing, we were consistent with [32,37,38,41,42] to ensure the fairness of model comparison. We explored two different training methods: one was the end-to-end training method, and the other was the two-stage training method. Experiments show that the two-stage training method can achieve better experimental results.

#### 4.2.1. End-to-End Training Method

The stochastic gradient descent (SGD) of the Nesterov momentum (0.9) is used as the optimization strategy. The setting of the loss function is shown in Equation (26). The weight decay was set to 0.0001, the batch size 32, and the initial learning rate 0.1. A total of 60 epochs were set in the NTU RGB+D 60 and NTU RGB+D 120 datasets, and the learning rate of the 30th and 40th epoch decreased by one-tenth, respectively. Seventy epochs were set in the Kinetics Skeleton 400 dataset, and the learning rates of the 45th epoch and the 55th epoch decreased by one-tenth, respectively.

#### 4.2.2. Two-Stage Training Method

In the first stage of training, the leading branch composed of multiple WGCN units was pre-trained and the batch size set to 64. $L_{w}$ in Equation (24) was used as the loss function for pre-training. Other training parameters not mentioned in the pre-training were consistent with the end-to-end method.

In the second stage of training, we first loaded the weight of the leading branch network from the pre-training and then trained the whole network. The L in Equation (26) was used as the loss function of the model. The batch size was set to 32, and the learning rate of the auxiliary branch network composed of multiple PGCN units stack was consistent with the end-to-end method, while the learning rate of the leading branch network for pre-training was set to one-tenth of the learning rate of the auxiliary branch. Other training parameters not mentioned were consistent with the end-to-end method.

### 4.3. Ablation Studies

Our ablation experiment was conducted on the X-Sub subset of the NTU RGB+D 60 dataset, and we tested the effectiveness of each component of our network and took the accuracy of recognition as the evaluation indicator.

#### 4.3.1. Symmetric Connection and Edge Connection

First of all, we evaluated the symmetric connection and edge connection components that we proposed to add to WGCN by using the WGCN single branch network. Table 1 shows the results of this ablation experiment. We used AAGCN [37] as the baseline method for WGCN. Due to the difference in hardware equipment used in training, the best recognition accuracy of our baseline network training in the X-Sub subset of the NTU RGB+D 60 dataset was 87.85%, which is slightly different from the recognition accuracy of 88.0% in the original paper.

Baseline+$A_{symmet}$ and baseline+$A_{edge}$ in Table 1 represent the addition of symmetric connection and edge connections to the baseline network, respectively. In WGCN, two kinds of connection methods are embedded. The recognition accuracy of the baseline model is improved by 0.63 percentage points for the single edge connection and 0.71 percentage points for the single symmetric connection, which shows that the two kinds of connections can effectively capture the potential relationship of each joint vertex and improve the efficiency of graph convolution. WGCN with both connections is 1.05 percentage points better than the baseline model, indicating that both connection modes can improve the performance of the model simultaneously.

#### 4.3.2. WPGCN and Two Training Methods

In Table 2, WGCN only contains the leading branch network, but not the auxiliary branch network, while WPGCN represents the whole network. The recognition accuracy obtained by the two different training methods is listed in Table 2. Obviously, the auxiliary branch network can significantly improve the accuracy of network recognition. Selecting the two-stage training method and end-to-end training method, the accuracy of WPGCN recognition is improved by 0.3 percentage points and 1.02 percentage points, respectively. The two-stage training method shows better performance than the end-to-end training method. Therefore, we chose to use the two-stage training method in the following other experiments.

#### 4.3.3. Adaptive Fusion Module

To test the effectiveness of the adaptive fusion module, we removed the adaptive fusion module from WPGCN. WPGCN (wo/AF) in Table 3 indicates the recognition accuracy without the adaptive module. It can be seen that the adaptive fusion module improves the recognition accuracy of the model by 0.26 percentage points. Comparative experiments were performed using constant parameters instead of learnable parameters of the adaptive fusion module. In the first experiment, the learnable parameters were set as constants: $\alpha_{1}$ = 0.2, $\alpha_{2}$ = 0.2, $\beta_{1}$ = 0.2, and $\beta_{2}$ = 0.2. In the second experiment, the learnable parameters were set as constants: $\alpha_{1}$ = 0.4, $\alpha_{2}$ = 0.4, $\beta_{1}$ = 0.4, and $\beta_{2}$ = 0.4. It can be seen from Table 3 that the adaptive fusion method can effectively improve the accuracy of the model compared with the constant parameters fusion method.

#### 4.3.4. Fusion Parameters λ

We set different fusion parameters λ in WPGCN for comparison experiments. We set seven groups of experiments at 0.1 intervals: λ = 0.5, 0.6, 0.7, 0.8, 0.9, 1.0, and 1.1. As shown in Table 4, when λ was set to 0.9, our model achieved the best recognition accuracy of 89.92%. In other experiments, we set λ to 0.9 without declaring the setting of λ to ensure the efficiency of recognition accuracy.

#### 4.3.5. Joint-Bone Two-Stream Fusion

We obtained the best performance with joint and bone feature fusion. In Table 5, Table 6 and Table 7, we verify the performance of our model in the joint bone fusion framework on three datasets, NTU RGB+D 60, NTU RGB+D 120, and Kinetics Skeleton 400, respectively. We list several state-of-the-art methods using the similar two-stream framework as a comparison.

### 4.4. Comparison against the State-of-the-Art

We compared the best performing model in the ablation study with several state-of-the-art methods on the NTU RGB+D 60, NTU RGB+D 120, and Kinetics Skeleton 400 datasets. The results are shown in Table 5, Table 6 and Table 7, respectively.

For the NTU RGB+D 60 dataset, our model achieved competitive performance on the X-Sub subset, reached the state-of-the-art level on the X-View subset, and significantly exceeded our baseline method. For the Kinetics Skeleton dataset, our model also surpassed state-of-the-art methods in both the top 1 and top 5 recognition accuracy indicators, and these two recognition accuracy indexes were more than 1% higher than MS-G3D, which is the most accurate method at present. For the NTU RGB+D 120 dataset, our model also outperformed state-of-the-art methods in both subsets X-Sub and X-Set.

In general, the recognition accuracy on three large-scale skeleton action recognition datasets proves that our model is highly competitive against others also considered in the state-of-the-art.

## 5. Conclusions

In this work, we propose a whole and part adaptive fusion graph convolutional network. WGCN is used to capture the whole scale skeleton features efficiently, and PGCN is used to obtain the unique features of each part of the human skeleton. The two kinds of features are adaptively fused many times to obtain more information and more comprehensive fusion features for the final recognition task. Experiments on three large datasets show that our model has more advantages than the existing models, and it also proves that the unique features of each part of the skeleton and the whole scale skeleton features can complement each other, thus helping in action recognition.

## Figures and Tables

**Table 1:** Comparison of the recognition accuracy of WGCN with or without the symmetric connection and edge connection on the NTU RGB+D 60 cross-subject (X-Sub) dataset. The best result is shown in bold.

Methods	Accuracy (%)
baseline (Js-AAGCN) [37]	88.0
baseline (we trained) [37]	87.85
baseline+$A_{symmet}$	88.48
baseline+$A_{edge}$	88.56
WGCN	**88.90**

**Table 2:** The accuracy of WPGCN is improved by the PGCN auxiliary branch and the comparison of recognition accuracy between the two training methods. The best result is shown in bold.

Methods	Accuracy (%)
WGCN	88.90
WPGCN (end to end)	89.20
WPGCN (two-stage)	**89.92**

**Table 3:** Comparison of the recognition accuracy with or without adaptive fusion and replacement of common fusion. The best result is shown in bold.

Methods	Accuracy (%)
WPGCN	**89.92**
WPGCN (wo/AF)	89.66
WPGCN (α, β = 0.2)	89.54
WPGCN (α, β = 0.4)	89.58

**Table 4:** Comparison of the recognition accuracy with different settings of the fusion parameters. The best result is shown in bold.

Methods	Accuracy (%)
WPGCN (λ=0.5)	89.59
WPGCN (λ=0.6)	89.75
WPGCN (λ=0.7)	89.80
WPGCN (λ=0.8)	89.88
WPGCN (λ=0.9)	**89.92**
WPGCN (λ=1.0)	89.91
WPGCN (λ=1.1)	89.86

**Table 5:** Recognition accuracy comparison against state-of-the-art methods on the NTU RGB+D 60 dataset. The best results are shown in bold.

Methods	NTU RGB+D 60		
	X-Sub (%)	X-View (%)	Reference
Lie Group [9]	50.1	52.8	CVPR2014
F2CSkeleton [23]	79.6	84.6	2018
SR-TSL [17]	84.8	92.4	ECCV2018
ST-GCN [31]	81.5	88.3	AAAI2018
AS-GCN [33]	86.8	94.2	CVPR 2019
2s-AGCN [32]	88.5	95.1	CVPR 2019
AGC-LSTM [39]	89.2	95.0	CVPR 2019
DGNN [38]	89.9	96.1	CVPR 2019
MS-AAGCN [37]	90.0	96.2	2019
SGN [46]	89.0	94.5	CVPR 2020
4sShift-GCN [42]	90.7	**96.5**	CVPR 2020
MS-G3DNet [41]	**91.5**	96.2	CVPR 2020
WPGCN (Joint Only)	89.9	95.8
WPGCN (Bone Only)	90.1	95.7
2s-WPGCN	91.1	**96.5**	Ours

**Table 6:** Recognition accuracy comparison against state-of-the-art methods on the NTU RGB+D 120 dataset. The best results are shown in bold.

Methods	NTU RGB+D 120		
	X-Sub (%)	X-Set (%)	Reference
2s-AGCN [32]	82.9	84.9	CVPR 2019
SGN [46]	79.2	81.5	CVPR 2020
4s Shift-GCN [42]	85.9	87.6	CVPR 2020
MS-G3D Net [41]	86.9	88.4	CVPR 2020
WPGCN (Joint Only)	83.7	85.8
WPGCN (Bone Only)	85.5	87.2
2s-WPGCN	**87.0**	**88.6**	Ours

**Table 7:** Recognition accuracy comparison against state-of-the-art methods on the Kinetics Skeleton 400 dataset. The best results are shown in bold.

Methods	Kinetics Skeleton 400		
	Top-1 (%)	Top-5 (%)	Reference
ST-GCN [31]	30.7	52.8	AAAI 2018
ST-GR [47]	33.6	56.1	AAAI 2019
AS-GCN [33]	34.8	56.5	CVPR 2019
2s-AGCN [32]	36.1	58.7	CVPR 2019
DGNN [38]	36.9	59.6	CVPR 2019
BAGCN [40]	37.3	60.2	2019
MS-AAGCN [37]	37.8	61.0	2019
MS-G3D Net [41]	38.0	60.9	CVPR2020
WPGCN (Joint Only)	38.1	60.7	
WPGCN (Bone Only)	37.1	60.1	
2s-WPGCN	**39.1**	**62.1**	Ours
